# Multimodal Large Language Models in Health Care: Applications, Challenges, and Future Outlook

**DOI:** 10.2196/59505

**Published:** 2024-09-25

**Authors:** Rawan AlSaad, Alaa Abd-alrazaq, Sabri Boughorbel, Arfan Ahmed, Max-Antoine Renault, Rafat Damseh, Javaid Sheikh

**Affiliations:** 1 Weill Cornell Medicine-Qatar, Education City Doha Qatar; 2 Qatar Computing Research Institute, Hamad Bin Khalifa University Doha Qatar; 3 Department of Computer Science and Software Engineering, United Arab Emirates University Al Ain United Arab Emirates

**Keywords:** artificial intelligence, large language models, multimodal large language models, multimodality, multimodal generative artificial intelligence, multimodal generative AI, generative artificial intelligence, generative AI, health care

## Abstract

In the complex and multidimensional field of medicine, multimodal data are prevalent and crucial for informed clinical decisions. Multimodal data span a broad spectrum of data types, including medical images (eg, MRI and CT scans), time-series data (eg, sensor data from wearable devices and electronic health records), audio recordings (eg, heart and respiratory sounds and patient interviews), text (eg, clinical notes and research articles), videos (eg, surgical procedures), and omics data (eg, genomics and proteomics). While advancements in large language models (LLMs) have enabled new applications for knowledge retrieval and processing in the medical field, most LLMs remain limited to processing unimodal data, typically text-based content, and often overlook the importance of integrating the diverse data modalities encountered in clinical practice. This paper aims to present a detailed, practical, and solution-oriented perspective on the use of multimodal LLMs (M-LLMs) in the medical field. Our investigation spanned M-LLM foundational principles, current and potential applications, technical and ethical challenges, and future research directions. By connecting these elements, we aimed to provide a comprehensive framework that links diverse aspects of M-LLMs, offering a unified vision for their future in health care. This approach aims to guide both future research and practical implementations of M-LLMs in health care, positioning them as a paradigm shift toward integrated, multimodal data–driven medical practice. We anticipate that this work will spark further discussion and inspire the development of innovative approaches in the next generation of medical M-LLM systems.

## Introduction

Large language models (LLMs) are sophisticated machine learning algorithms designed to process, understand, and generate humanlike language, enabling key developments in applications such as automated conversation, text analysis, creative writing, and complex problem-solving [1]. In health care, LLMs have shown remarkable potential, primarily through their ability to process and analyze textual content [2,3]. These models play a crucial role in assisting with diagnoses as they can efficiently process extensive textual patient histories and vast medical literature, providing clinicians with valuable insights [4-7]. However, most current LLMs are primarily limited to processing and generating textual content. While this unimodal focus on text-based operation has been transformative in the medical field, it does not fully capture the complex and diverse nature of health care practice [8].

In health care, diagnosing and treating a patient often involves a health care professional engaging in a comprehensive approach: listening to the patient, reviewing their health records, examining medical images, and analyzing laboratory test results—and all this over time. This multidimensional process exceeds the capabilities of current unimodal LLM systems. Moreover, nontextual data types play a crucial role in diagnosis, effective treatment planning, research, and patient care [9-11]. Such data may include medical imaging (eg, x-rays, magnetic resonance imaging [MRI], computed tomography [CT] scans, positron emission tomography scans, and pathology slides), electrophysiological data (eg, electrocardiography, electroencephalography (EEG), and electromyography), sensory data (eg, data from sensors of medical devices, such as pacemakers and continuous glucose monitors), videos (eg, recordings of surgeries, procedures, and patient interactions), omics data (eg, genomics, proteomics, metabolomics, and transcriptomics), and audio data (eg, recordings of patient interviews and heart and respiratory sounds).

The introduction of LLMs has been a key development in the field of artificial intelligence (AI) and natural language processing (NLP). In 2010, the emergence of deep learning revolutionized LLMs. Recurrent neural networks (RNNs), particularly long short-term memory (LSTM) networks [12], allowed models to better capture sequential data and context. However, the major breakthrough occurred in 2017 with the introduction of transformer models [13], which are widely used for NLP tasks. A transformer is a type of neural network architecture that uses a self-attention mechanism to capture long-range dependencies between words in a sentence. While the computation in architectures such as RNNs and LSTM networks is sequential and slow for long sequences [14], self-attention can be parallelized and made highly scalable. Transformers have been widely trained using 2 objectives. The first objective is mask language modeling (MLM), where the objective is to learn text reconstruction by randomly masking several words in text (eg, 10%) and update the transformer weights toward this goal. Encoder transformers such as Bidirectional Encoder Representations From Transformers (BERT) [15] have been trained with the MLM objective. The second widely used objective is the next word prediction or causal language modeling. The self-attention mechanism is masked such that, at each position in the sequence, the model is able to attend only to the left words. This modeling approach mimics how text is read by humans in one direction. The self-attention mechanism allows for the computation of the probability of predicting the next word in a document by attending to the most relevant parts of the input sequence [13,16]. By applying the prediction autoregressively, the transformer model performs a text completion task by generating multiple words. Interestingly, transformers extend beyond just handling natural language data. They can effectively compute representations for various data types provided these can be represented as a sequence of tokens. The letters are elementary entities that constitute the sequences. The unique set of tokens represents the vocabulary. For example, in DNA sequence, each nucleotide could be represented by tokens from the vocabulary of 4 tokens: A, C, G, and T. This capability includes processing elements such as video frames, audio spectrograms, time-series data, code snippets, or protein sequences. BERT [15] is among the first major models to use transformers. Subsequently, a series of medical BERT models were proposed to accelerate medical research [6,17-20].

In 2022, OpenAI released ChatGPT (GPT-3.5), a significant iteration in the generative pretrained transformer (GPT) series [21]. As an LLM, ChatGPT has been trained on a vast collection of text data, which enables it to generate humanlike responses across a broad spectrum of topics and formats. ChatGPT has also shown its potential to become a valuable resource in health care, making significant contributions to various medical applications. It provided opportunities for advancing diagnostic accuracy, personalized treatment planning, and medical research, as well as optimizing health care administration and enhancing communication in patient care [22-28]. In addition, several open-source LLMs such as LLaMA [29], Flan-T5 [30], Vicuna [31], and Alpaca [32] have substantially driven progress and contributed to the field of LLMs. Although these LLM systems have achieved considerable success, they are predominantly limited to single data types. This limitation makes them less effective for the multimodal nature of medicine, where handling multiple data types is often required. Therefore, considerable efforts have been dedicated to creating LLMs that handle multimodal inputs and tasks, ultimately leading to the development of multimodal LLMs (M-LLMs). In 2023, OpenAI released GPT-4, an M-LLM with the dual capability to process and respond to both text and images. Following the release of GPT-4, several medically adapted versions of this model have been developed [33-37]. These specialized versions of GPT-4 have been tailored to interpret medical data, understand patient queries, and assist in diagnostic processes using both text and image modalities. Building on these insights, M-LLMs are increasingly recognized as systems capable of integrating various data types to facilitate comprehensive patient assessments, ensuring accurate diagnoses. In addition, they hold the potential to streamline operations, significantly improving efficiency in both clinical and administrative tasks. Most importantly, with appropriate oversight, M-LLMs could provide personalized care by tailoring treatment plans to meet the individual needs of patients, thereby enhancing the quality of health care services.

Recent studies [38,39] have explored the capabilities of M-LLMs within the health care sector. However, these studies exhibit several limitations. First, the range of data modalities examined is often restricted to text, images, videos, and audio [38], with some studies focusing narrowly on a limited number of clinical applications [39]. Second, the discussion regarding the potential uses of M-LLMs in health care is largely theoretical [38], leading to a significant gap in demonstrating their application in actual health care environments. Third, although the challenges of integrating diverse data types into M-LLMs are recognized, there is limited exploration of possible solutions or ongoing research aimed at overcoming these technical barriers [38,39].

This paper aims to present a detailed, practical, and solution-oriented perspective on the use of M-LLMs in the medical field. We unify the discussion by focusing on how M-LLMs can serve as a transformative tool that integrates various data modalities to enhance health care outcomes. Specifically, we aim to (1) broaden the analysis of M-LLM applications in health care to include additional data modalities, such as time-series data and omics data, alongside conventional modalities such as images, text, audio, and video; (2) highlight practical examples in which M-LLMs have been or could be effectively applied in health care settings; (3) outline current technological advancements to address the technical and ethical challenges; and (4) propose future research directions to fully exploit the capabilities of M-LLMs. Our unique contribution lies in providing a comprehensive framework that links these diverse aspects, offering a unified vision for the future of M-LLMs in health care.

## Background

### Multimodal Learning

In the context of M-LLMs, the term *multimodal* encompasses a range of scenarios in data processing and interpretation. First, it refers to LLMs in which the input and output to the system involve different modalities, such as text-to-image or image-to-text conversions. Second, it describes LLM systems capable of handling inputs from multiple modalities, such as those that can process both text and images simultaneously. Finally, multimodality characterizes systems designed to generate outputs in >1 modality, such as systems capable of producing both textual and image-based content [40].

Several previous works have developed basic M-LLMs by aligning the well-trained encoders from different modalities with the textual feature space of LLMs. This approach enables LLMs to process inputs other than text, as seen in various examples [41-44]. For instance, Flamingo [45] uses a cross-attention layer to link a frozen image encoder with LLMs. LLaVA [46] uses a basic projection method to incorporate image features into the word embedding space. Similarly, models such as Video-Chat [47] and Video-LLaMA [48] are designed for video comprehension, whereas SpeechGPT [49] is tailored for audio processing. A notable example is PandaGPT [50], which uniquely manages to interpret 6 different modalities at the same time, achieved through the integration of a multimodal encoder known as ImageBind [51].

Despite numerous efforts focusing on understanding multimodal content at the input side, there is a significant gap in the ability to produce outputs in various modalities beyond textual content. This underscores the importance of developing any-to-any M-LLMs, which are crucial for realizing real artificial general intelligence (AGI) [52,53]. Such models should be capable of receiving inputs in any form and providing responses in the appropriate form of any modality.

### From Unimodal Limitations to Multimodal Solutions

**Figure 1:**
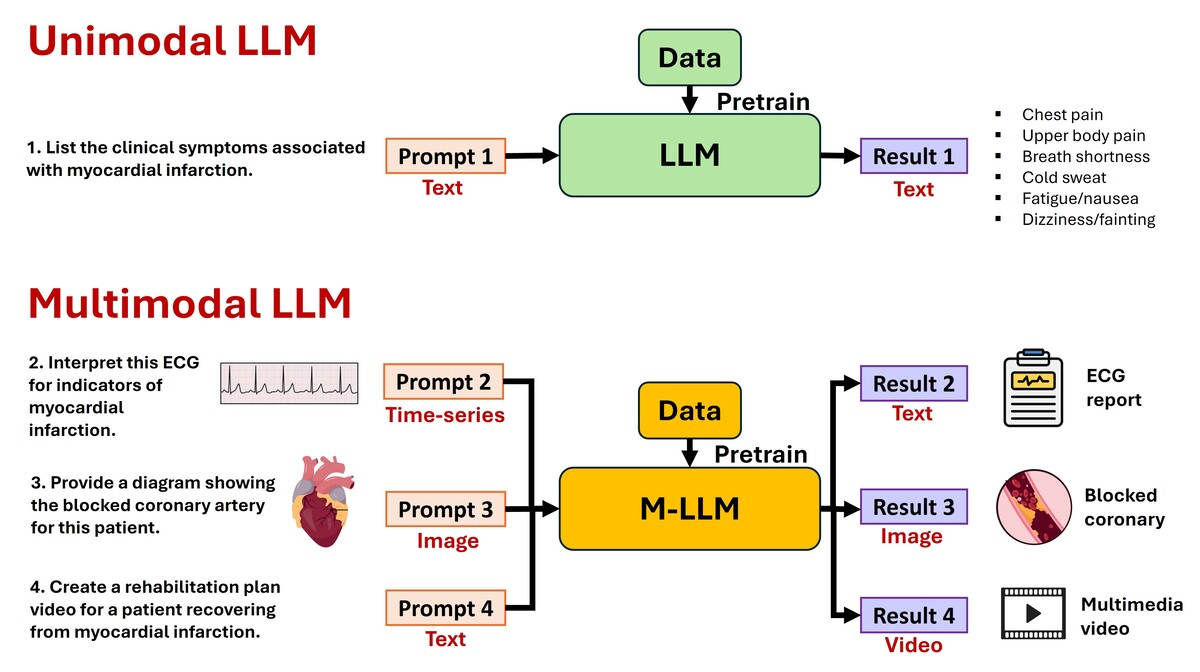
Unimodal large language model (LLM) and multimodal LLM (M-LLM) examples. ECG: Electrocardiogram.

Unimodal LLMs generate content in the same modality as that in which they receive inputs, typically text, whereas M-LLMs are capable of processing inputs from various modalities and delivering outputs across multiple modalities, as illustrated in Figure 1. Despite their remarkable abilities, unimodal LLMs in medicine have inherent limitations that can be effectively overcome by shifting toward multimodal systems. In Table 1, we summarize these limitations in the medical field and illustrate how the integration of a multimodal approach can address these challenges.

**Table 1:** Summary of unimodal (text) large language model (LLM) limitations in medicine and potential multimodal LLM solutions.

Unimodal (text) LLM limitation	Description of unimodal limitation	Multimodal LLM solution	Description of multimodal solution
Lack of diagnostic imaging context	Unimodal LLMs in medicine can only process textual patient data and cannot interpret diagnostic images, which are vital in many clinical scenarios.	Integration of diagnostic imaging data	Multimodal models process and integrate diagnostic imaging information (eg, x-rays and MRIs^a^), improving diagnostic accuracy and patient outcomes.
Inability to analyze temporal data	Text LLMs often struggle with interpreting time-series data, such as continuous monitoring data or progression of diseases, which are vital for tracking patient health over time.	Time-series data integration	Multimodal systems incorporate and analyze temporal data, such as ECG^b^ readings or continuous monitoring data, enabling dynamic tracking of patient health and disease progression.
Absence of auditory data interpretation	Unimodal LLMs grapple with audio analysis, which limits their effectiveness in health care applications that rely on processing spoken interactions or auditory signals.	Audio data processing	Multimodal systems can process and understand audio signals, such as patient verbal descriptions and heartbeats, enhancing diagnostic precision.
Limited comprehension of complex medical scenarios	Unimodal LLMs struggle with interpreting complex medical conditions that require a multisensory understanding beyond text.	Multisensory data integration	By processing clinical notes, diagnostic images, and patient audio, multimodal systems offer more comprehensive analyses of complex medical conditions.
Overfitting to clinical textual patterns	Sole reliance on clinical texts can lead LLMs to overfit to textual anomalies, potentially overlooking critical patient information.	Diverse clinical data sources	Diversifying input types with clinical imaging and audio data allows multimodal systems to increase the number of training data points and, hence, reduce overfitting, enhancing diagnostic reliability.
Bias and ethical concerns	Unimodal LLMs, especially text-based ones, can inherit biases and misconceptions present in their training data sets, affecting patient care quality.	Richer contextual patient data	Multimodal systems use diverse modalities, including patient interviews and diagnostic images, to provide a broader context that can mitigate biases in clinical decision-making.

^a^MRI: magnetic resonance imaging.

^b^ECG: electrocardiography.

## Foundational Principles of M-LLMs

### Overview

**Figure 2:**
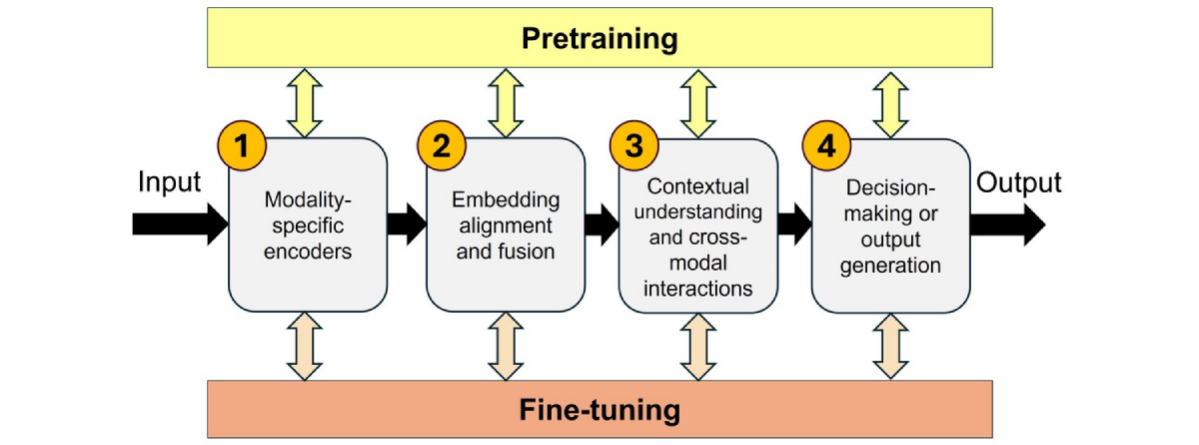
Main components of multimodal large language model training.

The field of M-LLMs is evolving rapidly, with new ideas and methodologies being continuously developed. The training of medical M-LLMs is a complex process designed to effectively integrate and interpret the diverse types of data encountered in clinical practice. Typically, the architecture of an M-LLM system encompasses four key stages (Figure 2): (1) modality-specific encoding, (2) embedding alignment and fusion, (3) contextual understanding and cross-modal interactions, and (4) decision-making or output generation. In addition to these stages, pretraining and fine-tuning processes play a crucial role, interacting with and enhancing each of the aforementioned stages.

This section presents the foundational principles that currently guide the development and functioning of medical M-LLMs. Importantly, the specific architecture of an M-LLM might vary significantly to meet particular requirements, such as the types of data it needs to handle, the tasks it is designed to perform, and the level of interpretability and performance required. Therefore, while the stages outlined provide a high-level overview of an M-LLM system’s architecture, actual implementations may vary widely to accommodate the unique demands of each application. As this field progresses, we anticipate that the guiding principles of medical M-LLMs will continue to be shaped by emerging ideas and technological advancements.

### Modality-Specific Encoding

The purpose of this stage is to transform raw data from each modality into a format that the model can understand and process. This involves using modality-specific encoders to encode various data types into rich and informative representations that subsequent components of the M-LLM architecture can effectively leverage. These modality-specific encoders are meticulously trained using extensive data sets of unlabeled information to generate embeddings that accurately encapsulate the data’s content. The encoders are trained in an unsupervised manner using a large collection of data sets. Selecting the appropriate encoding architecture and optimizing the training methodology are imperative and often tailored to the specific characteristics of the data and the requirements of the medical task at hand. For example, image encoders (eg, transformers [54] and convolutional neural networks (CNNs) [55,56]) are designed to capture fine-grained patterns or anomalies crucial for diagnosis, whereas text encoders (BERT [15]) aim to comprehend complex medical terminology and patient histories. Similarly, audio encoders (such as WaveNet [57] and DeepSpeech [58]) are optimized to distinguish subtle variations in sounds, such as differentiating between normal and abnormal heart or lung sounds. Time-series encoders (such as transformer-based models [15,59-61] and LSTM [12]) are intended to detect critical changes over time, signaling the need for urgent medical intervention. Finally, omics encoders (eg, DeepVariant [62], Basenji [63], and DeepCpG [64]) focus on identifying genetic markers or patterns associated with specific diseases, aiding in the development of targeted therapies.

### Embedding Alignment and Fusion

The purpose of this stage is to harmonize the embeddings from different modality-specific encoders into a combined representation that reflects the combined information from all input modalities. This might involve techniques such as concatenation [65] and attention mechanisms [13] or more sophisticated methods such as cross-modal attention [66,67] and tensor fusion [68]. While modality-specific encoding relies solely on unsupervised data, embedding alignment needs annotated data across modalities. Moreover, the alignment mechanism in medical M-LLMs may require incorporating domain-specific knowledge to enhance its understanding and integration of medical data. For example, it might use known relationships between symptoms and diseases or anatomical correlations to better align and interpret the multimodal data. This results in a more accurate, reliable, and clinically relevant synthesis of information.

### Contextual Understanding and Cross-Modal Interactions

The objective of this stage is that the M-LLM not only comprehends the individual modalities but also discerns their interrelations and collective contributions to the overall medical analysis or diagnostic task. This necessitates the deployment of advanced neural network architectures, notably, transformers equipped with cross-modal attention mechanisms [66,67]. These mechanisms enable the M-LLM to dynamically prioritize and integrate features across different modalities, enhancing its ability to make informed medical decisions. In addition, attention-based fusion strategies [68] could be implemented to weigh and integrate information from disparate sources, adjusting the focus of the model according to the contextual relevance of each data point from each modality.

### Decision-Making or Output Generation

This component is the actionable end of the model that produces the final output or decision based on the integrated and interpreted multimodal data. This could be a classification layer capable of distinguishing between different medical conditions or a sequence generator for creating detailed medical reports. When encoder architectures are used, the model head layer can be trained for downstream classification tasks. When decoder architectures are used, the model head layer outputs logits of vocabulary tokens that can be applied in an autoregressive manner to synthesize a response. For instance, in diagnostic imaging, the model might analyze combined textual and visual embeddings to identify and categorize pathologies. In treatment recommendation systems, the model could synthesize patient history, current symptoms, and laboratory test results to suggest personalized treatment plans. The effectiveness of this stage depends on the precision of the previous components.

### Pretraining and Fine-Tuning

Pretraining and fine-tuning are fundamental processes in the development and optimization of LLMs, including multimodal ones [69]. They are not just single steps but integral, ongoing processes that influence and enhance all components of an M-LLM system’s architecture. They interact with the 4 previous components of the M-LLM architecture in multiple ways.

Pretraining begins with modality-specific encoders, focusing on learning general features and representations for each modality. For instance, encoders are pretrained on large data sets to understand text, images, or audio before they are combined or applied for specific tasks. Within the embedding alignment and fusion component, pretraining enables models to learn preliminary methods for aligning and integrating embeddings from different modalities, especially in unsupervised or self-supervised setups in which the model is exposed to vast amounts of multimodal data. In the context of understanding and cross-modal interactions, pretraining lays the foundation for learning complex relationships between modalities. As the model is exposed to a wide and varied range of multimodal data, it learns to identify common patterns and interactions. Although pretraining does not directly result in final decisions or outputs for the decision-making or output generation component, it establishes essential capabilities and knowledge. This foundational understanding equips the model to later perform specific tasks more effectively.

Fine-tuning adapts a pretrained model to downstream tasks or data sets. It involves adjusting and optimizing the model’s parameters and architecture components using a smaller, more task-specific data set. The fine-tuned models are capable of following instructions and responding to questions and queries. In the context of M-LLMs, fine-tuning would adjust how individual modalities are encoded, how they are aligned and fused, and how the model makes decisions based on this refined understanding.

## Applications

### Overview

**Figure 3:**
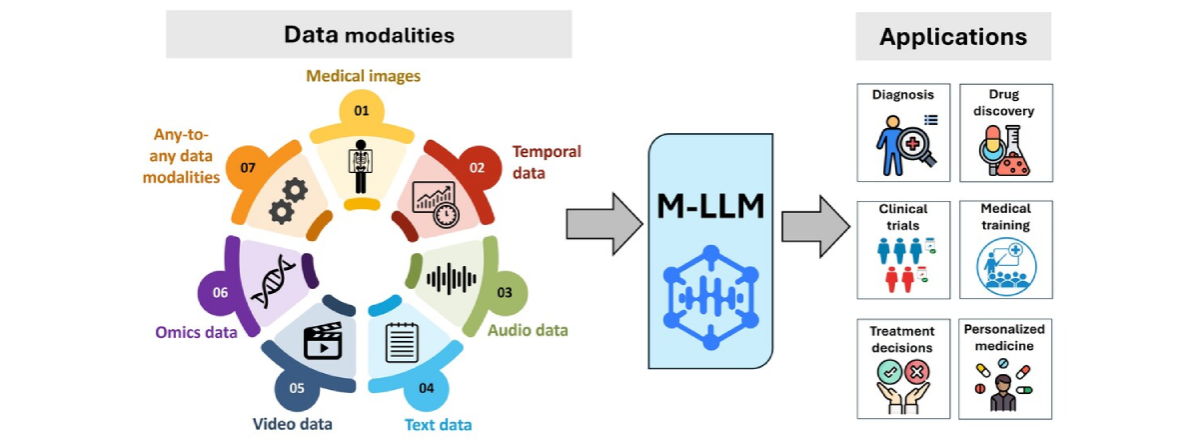
Applications of multimodal large language models in health care.

M-LLMs hold transformative potential for numerous medical applications, demonstrating unparalleled proficiency in processing and integrating diverse data types, as shown in Figure 3. In this section, we discuss the applications of M-LLMs in clinical practice organizing them according to data type. These categories include medical images, temporal data (encompassing time-series and event data), audio, video, text, omics data, and any-to-any M-LLMs. This structured approach enables a thorough exploration of how these models can revolutionize health care practices based on their ability to synthesize and analyze complex multimodal information.

### Medical Images

M-LLMs, equipped with advanced capabilities to process and interpret various image modalities, can significantly enhance diagnostic accuracy and efficiency in medical imaging applications. Examples of these image modalities include x-rays, MRI scans, CT scans, positron emission tomography scans, ultrasound images, digital pathology slides, and retinal images. Each modality provides unique insights into the body’s internal structures, facilitating comprehensive analysis and aiding in the early detection, diagnosis, and monitoring of diseases. For example, in radiology, M-LLMs are instrumental in analyzing CT and MRI images to offer precise, quantifiable data for identifying and characterizing anomalies such as tumors, fractures, and signs of chronic diseases. In addition, these models support the generation of automated radiological reports that summarize findings and suggest potential diagnoses. It is also possible to use M-LLM embedding to retrieve similar cases based on keyword searching. Conversely, M-LLMs allow for the annotation and tagging of medical images with keywords. This enables additional analytics applications. Similarly, in pathology, M-LLMs interpret tissue sample slides, identifying disease markers that are often subtle and challenging to discern. In dermatology, M-LLMs apply their image analysis processes to assess photos of skin lesions, aiding in the early detection of skin cancers such as melanoma [70].

Significant progress has been made in the field of general-domain image-text M-LLMs through the development of models such as GLaMM [71], Qwen-VL [72], SpatialVLM [73], InternVL [74], Osprey [75], Vary [76], ShareGPT4V [77], OtterHD [78], LION [79], SPHINX [80], BLIVA [81], SVIT [82], LLaVA [46], and CoVLM [83]. These advancements have been made possible by leveraging billions of image-text pairs predominantly sourced from the public web, enabling these models to analyze and integrate visual and textual information to understand and generate complex and contextually relevant responses. Such M-LLMs with vision capabilities can be adapted for medical imaging applications (eg, LLaVA-Med [84], PMC-VQA [85], Med-Flamingo [86], and PeFoMed [87]). However, an important question arises regarding whether such general-domain models can deeply understand medical images or whether they simply recognize superficial patterns from extensive pretraining. Previous work [88] evaluated the performance of a general-domain M-LLM in biomedical image classification tasks. The study aimed to determine whether such M-LLMs can develop usable internal representations of medical images and whether these representations could effectively distinguish between various medical subclasses. The results showed that generalist models can inherently understand medical images and, in some medical contexts, even outperform specialized, task-specific pretraining methods. Therefore, using representations from generalist models may offer a data-effective solution for developing classification models in the medical imaging domain.

### Temporal Data

M-LLMs with the ability to process and interpret time-stamped sequences of data offer significant potential in areas such as real-time patient status tracking in intensive care units, longitudinal studies for chronic disease management, and predictive analytics for patient risk assessment. M-LLMs designed with temporal dimensions acquire predictive capability and skills in extrapolating the understanding of medical conditions over time. Temporal data include time-series, spatiotemporal, and event data. For the purpose of this paper, our focus will be on time-series and event data.

Time-series data are a sequence of data points collected or recorded at regular time intervals, with each data point being time-stamped. Examples include a patient’s heart rate recorded over time and continuous glucose monitoring (CGM). In critical care settings, M-LLMs can detect early signs of clinical deterioration, such as sepsis or cardiac events, from continuous monitoring of vital signs. In neurology, M-LLMs process EEG data to detect neurological anomalies, such as seizure patterns.

Event data are a record of discrete actions or occurrences at specific time points or over intervals. Unlike time-series data, they do not have to be regularly timed. Examples include electronic health records (EHRs) detailing various discrete events in a patient’s medical history, such as physician visits, hospital admissions, and prescription records, or sensor data recording specific occurrences, such as motion sensors being triggered with movement. Each event is time-stamped but does not occur at regular intervals. M-LLMs are instrumental in extracting meaningful insights from EHRs, which encompass diverse and nonregularly timed medical events [89]. M-LLMs can analyze the sequence and context of these events, providing a comprehensive understanding of a patient’s medical history. This analysis can lead to more accurate diagnoses, tailored treatment strategies, and improved management of chronic conditions. In addition, M-LLMs can process sensor data, such as motion sensor activations in older adult care settings, offering real-time insights into patient activity and well-being.

Significant advancements have been made in M-LLMs with temporal analysis capabilities, including models such as Time-LLM [90], LLM4TS [91], TEMPO [92], and PromptCast [93], among others [94,95]. However, there is still a lack of M-LLMs specifically designed for medical temporal data. Some of the existing M-LLMs with temporal capabilities could be adapted for medical applications [89,96], or new models specifically designed and pretrained on medical temporal data can be developed.

### Audio

Medical M-LLMs that can process and comprehend audio signals have the potential to significantly enhance health care. These models can analyze vocal patterns and breathing sounds to identify respiratory conditions such as asthma or chronic obstructive pulmonary disease (COPD) early in their development. In addition, M-LLMs can be used in mental health to detect subtle changes in speech patterns, affective tone, and vocal tone that may indicate depression, anxiety, or stress, offering a noninvasive diagnostic tool that complements traditional assessment methods. Moreover, audio-based M-LLMs facilitate continuous monitoring of patients in intensive care unit settings, using sound analysis to alert medical staff to changes in patient condition that might necessitate immediate intervention. Furthermore, these models enhance patient engagement and education by converting medical advice into accessible audio formats tailored to individual patient needs and comprehension levels. They can also aid in the early detection of neurological disorders through speech irregularities, help monitor sleep apnea by analyzing breathing patterns during sleep, and support speech therapy for stroke survivors by tracking progress in speech fluency and pronunciation.

Numerous audio-text M-LLMs, leveraging transformer-based architectures, have integrated text- and audio-based language models, such as AudioPaLM [97], AudioLM [98], Pengi [99], AudioGPT [100], SpeechGPT [49], VioLA [101], and SALMONN [102], into a unified multimodal architecture. This architecture is capable of processing and generating both text and speech, facilitating applications such as speech recognition and speech-to-speech translation. However, there is a gap in the development of large audio models specifically tailored for medical applications [103]. Nonetheless, these existing M-LLMs with audio capabilities may be adapted and refined to address the requirements of medical-related tasks.

### Text

Although text-based LLMs are not inherently multimodal, integrating text with other data modalities such as images and audio transforms them into the core of M-LLMs. In clinical practice, these text-based components of M-LLMs can be applied in several ways. For instance, they facilitate the automated generation of patient reports by interpreting and summarizing complex medical language and data, including diagnostic imaging and laboratory test results. M-LLMs with additional skills in understanding tabular and other structured textual data are expected to perform better on EHR data. Furthermore, text M-LLMs play a crucial role in analyzing the large volumes of clinical notes routinely available in EHRs to predict clinical outcomes. In addition, they enhance medical education and training by providing simulations and interactive learning experiences based on extensive medical literature and case studies.

There is a growing interest in the development of M-LLMs that incorporate text data, demonstrating the vast potential and ongoing innovations in this field. Examples of biomedical text LLMs include BiMediX [104], BioBERT [105], PubMedBERT [106], and ClinicalBERT [20]. BioBERT is a biomedical language representation model designed for text mining tasks such as named entity recognition, relation extraction, and question answering in the biomedical domain. PubMedBERT is specifically pretrained from scratch on PubMed articles, ensuring a highly focused approach to understanding medical literature. ClinicalBERT is a BERT model pretrained on generic EHR clinical documentation and discharge summaries. BiMediX is the first bilingual medical LLM with expertise in both English and Arabic, facilitating several medical interactions, including multiturn conversations, multiple-choice queries, and closed question answering.

### Videos

M-LLMs hold significant promise in transforming the analysis and interpretation of various types of video data within medical settings. In surgical training, M-LLMs can analyze and interpret surgical videos, providing real-time feedback and educational insights. In physical therapy, M-LLMs can analyze patient movement videos, aiding in designing targeted rehabilitation programs and monitoring patient progress. They can also be used in psychiatric evaluations to assess behavioral patterns through video assessments. Furthermore, M-LLMs can be used in internal examinations, interpreting recordings from endoscopic and laparoscopic procedures to identify abnormalities and support real-time decision-making during these procedures. Their applications extend to home health care, allowing for remote patient monitoring through video to track well-being. They are also used in sleep studies, where video recordings assist in diagnosing disorders such as sleep apnea. In dermatology, video analysis of skin conditions over time helps in tracking disease progression.

The progress in M-LLMs for video data analysis, demonstrated by models such as Video-Chat [47], Video-ChatGPT [107], Video-LLaMA [48], LLaMA-VID [108], MotionGPT [109], LAVENDER [110], MovieChat [111], Vid2Seq [112], VideoLLM [113], and VTimeLLM [114], shows significant promise for the development of models tailored to medical applications. The success of these models in nonmedical settings lays a foundation for similar advancements in the health care sector. However, a critical aspect in applying these models to medicine is the incorporation of domain-specific medical knowledge. Medical videos require not just technical analysis but also contextual interpretation aligned with patient history, presenting symptoms, and potential diagnoses. Furthermore, the operational demands of these models in clinical environments are stringent. They must function in real time or near real time to offer actionable insights during critical medical procedures, such as providing alerts during surgeries or continuous patient monitoring.

### Omics Data

M-LLMs leveraging omics data, encompassing genomics, transcriptomics, proteomics, and other omics technologies, have the potential to significantly enhance personalized medicine and clinical diagnostics. By integrating and interpreting complex omics data sets, M-LLMs can uncover novel biomarkers for diseases, predict patient responses to specific treatments, and facilitate the development of targeted therapies. For example, in oncology, these models can analyze genetic mutations and expression patterns to guide cancer treatment strategies. Similarly, in cardiology, omics data analysis can help identify genetic risk factors for heart diseases, enabling preventative interventions. M-LLMs also support drug discovery processes by predicting the efficacy and side effects of potential drugs based on the omics profiles of diverse patient populations.

Several M-LLMs have been developed using omics data for a wide range of biomedical applications [115]. In genomics, DNA sequence language models are used for a variety of predictive tasks. These tasks include predicting genome-wide variant effects (GPN [116]; DNABERT [117]; and its subsequent evolution, DNABERT-2 [118]), predicting DNA cis-regulatory regions (DNAGPT [119], DNABERT, and DNABERT-2), predicting DNA-protein interactions (TFBert [120] and MoDNA [121]), and determining RNA splice sites from DNA sequences (DNABERT and DNABERT-2). In transcriptomics, RNA sequence language models are used for RNA splicing prediction (SpliceBERT [122]), assessment of long noncoding RNAs’ coding potential (LncCat [123]), RNA-binding protein interactions (BERT-RBP [124]), RNA modification identification (BERT-m7G [125]), and predictions related to protein expression and messenger RNA degradation (CodonBERT [126]). In proteomics, protein language models are used for secondary structure and contact prediction (ProtTrans [127]), protein sequence generation (ProGen [128]), protein function prediction (ProtST [129]), major posttranslational modification prediction (ProteinBERT [130]), biophysical property prediction (PromptProtein [131]), and advancing the state of the art in proteomics [132,133].

### Any-to-Any M-LLMs

Current M-LLMs are primarily limited to multimodal comprehension on the input side, possessing limited capabilities to generate content across various modalities [134,135]. Given that clinicians frequently interact and communicate using a variety of medical modalities, the potential applications of any-to-any M-LLMs, which can accept input in any modality and produce output in any modality, are numerous. For instance, clinicians can provide a combination of textual patient history, radiographic images, and audio recordings of patient symptoms as input to the M-LLM. The M-LLM could then analyze this multimodal input to diagnose the patient’s condition. Subsequently, it could generate a multimodal output that includes a textual report summarizing the diagnosis, annotated images highlighting areas of concern, and an audio explanation that can be easily shared with patients or other medical professionals.

There is an increasing interest in the development of any-to-any M-LLMs, highlighting the significant potential of their applications across various domains. For instance, NExT-GPT [136] enhances an LLM with multimodal adapters and a range of diffusion decoders, enabling the model to process and generate outputs in any combination of text, images, videos, and audio. Macaw-LLM [137] integrates images, audio, and textual data using 3 primary components: a modality module for encoding multimodal data, a cognitive module for leveraging pretrained LLMs, and an alignment module for synchronizing diverse representations. OneLLM [138] incorporates 8 unique modalities within a single framework using a multimodal alignment pipeline, which can be further expanded to include additional data modalities. These models, among others [139,140], can be tailored and fine-tuned to specifically address the unique demands of tasks related to health care.

### Use Case Example

In this section, we present a use case that demonstrates the practical application of M-LLMs in health care using the Contrastive Learning From Captions for Histopathology (CONCH) model [141]. CONCH is a vision-language M-LLM specifically designed for computational histopathology. It is pretrained on the largest histopathology-specific vision-language data set, enabling it to create effective representations for non–H&E (hematoxylin and eosin)-stained images, such as immunohistochemistry and special stains, without relying on large public histology slide collections such as The Cancer Genome Atlas, Pancreatic Cancer AI Platform, and Genotype-Tissue Expression.

**Figure 4:**
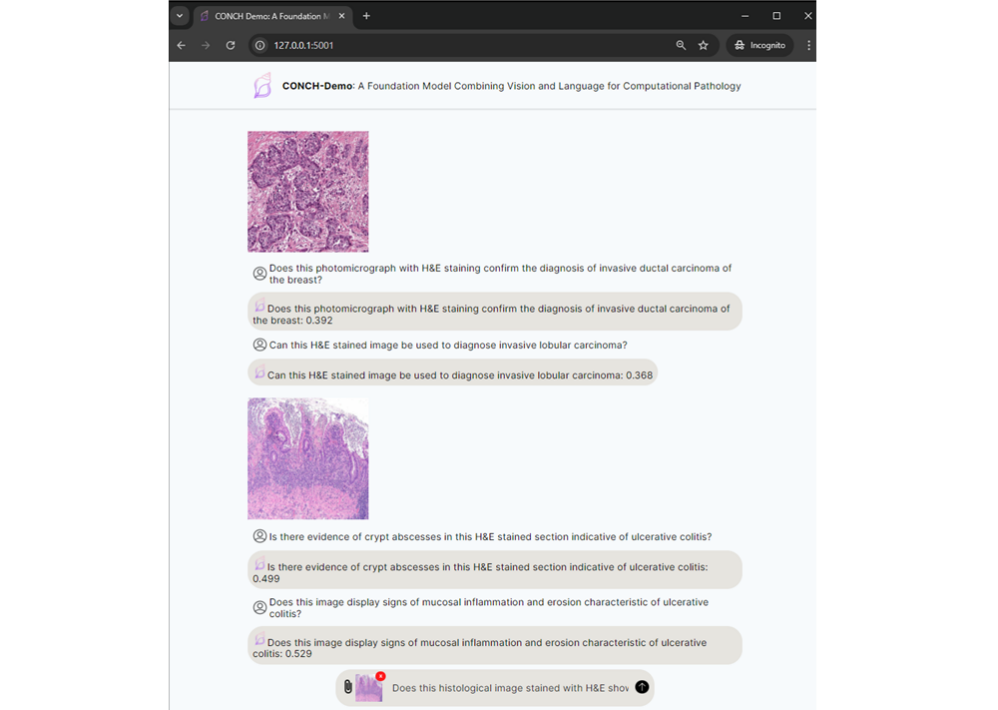
Demonstration of the Contrastive Learning From Captions for Histopathology (CONCH) model as a text-vision foundation model for histopathology analysis.

For this experiment, we used the pretrained model weights available on Hugging Face [141] and installed the CONCH package from the official repository [142]. The experiment was conducted on a Linux machine equipped with an NVIDIA GeForce GTX 1080 Ti graphics card using a web-based demonstration application developed using the Flask web framework. The application created a ChatGPT-like interface for zero-shot cross-modal retrieval, accepting both pathology-related text prompts and pathological images. It computed cosine similarity and provided retrieval scores based on the input data. Figure 4 illustrates how CONCH was used to analyze 2 histopathology slides, providing confidence scores for various diagnostic questions. The model processes both the images and corresponding text prompts, offering a zero-shot cross-modal retrieval approach to assist in diagnosing conditions such as invasive ductal carcinoma, invasive lobular carcinoma, and ulcerative colitis.

This use case example highlights the potential of M-LLMs such as CONCH to enhance computational pathology by enabling advanced, multimodal data retrieval and analysis even in complex and specialized medical imaging tasks.

## Challenges

### Overview

**Figure 5:**
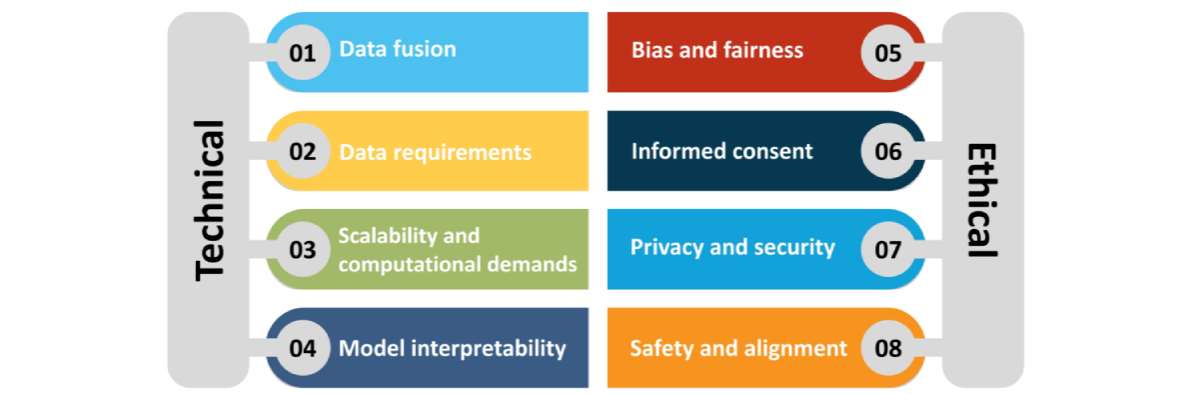
Challenges of multimodal large language models in health care.

While the potential of M-LLMs is promising, it is crucial to understand the significant technical and ethical challenges and limitations that accompany their development and deployment in health care (Figure 5). From a technical perspective, challenges include integrating diverse data sources (data fusion), meeting extensive data requirements, ensuring scalability and managing computational demands, and improving the interpretability of M-LLMs. Ethically, issues such as bias and fairness, obtaining informed consent, data privacy and security, and the safety and alignment of these models in clinical practice present substantial obstacles. In this section, we discuss these challenges and propose potential solutions to tackle them.

### Technical Challenges

#### Data Fusion

##### Problem

Data fusion in medical M-LLMs is a sophisticated and complex process that requires the integration of heterogeneous data types to create a comprehensive and multidimensional representation of patient health. This integration process encompasses several technical challenges that must be adeptly managed. The first challenge is the temporal and spatial alignment of different data modalities, where aligning data from diverse sources such as medical images, videos, and text-based records is crucial to ensure that all data points are synchronized and that temporal data (showing changes over time) and spatial data (showing anatomical or physiological details) are correctly correlated. Second, handling data sparsity and missingness is vital as it can significantly impact diagnosis and treatment. For example, missing frames in a medical video could miss critical changes in a patient’s condition, incomplete medical images may not fully reveal the extent of a disease, and gaps in EHRs can result in a lack of historical context for patient care, necessitating sophisticated techniques to infer missing information without compromising diagnostic accuracy. Furthermore, normalization and standardization are essential given the varied formats, scales, and resolutions of different data modalities, for example, adjusting the scale of medical images to a standard range, normalizing text data from clinical notes to a uniform format for analysis, and standardizing video data to ensure consistent frame rates and resolutions. These challenges highlight the complexity of integrating diverse data types used in M-LLMs, underscoring the need for advanced computational techniques and algorithms to address these issues effectively.

##### Potential Solution

Beyond foundational methods for data fusion, a variety of advanced techniques exist that can enable M-LLMs to more effectively integrate different modalities. Prompt-based multimodal fusion [143] is one such framework that enables bidirectional interaction among different modalities through a 2-stream structure, typically involving parallel construction of the multimodal model through pretrained language and image models. Hybrid fusion [144] integrates unstructured and structured data along with other multimodal sources via a pretrained language model, capturing a more comprehensive patient representation. Gated fusion [145,146] uses mechanisms such as neural network gates or attention mechanisms to dynamically emphasize or de-emphasize different aspects or modalities of the data based on the context. Finally, tensor fusion [68] constructs a higher-order tensor representing all feature combinations across modalities, which is then decompressed or factorized to a lower dimension for tractable computation while preserving the depth of multimodal interactions.

#### Data Requirements

##### Problem

In the pretraining phase of M-LLMs, large and diverse data sets with extensive labeling in many cases are required to capture a wide range of general knowledge across different modalities (eg, text, images, and audio). The primary goals of pretraining are to develop robust feature representations and ensure that the model can handle the inherent variability in real-world data. However, such multimodal medical data sets are currently limited, and the acquisition of such large-scale labeled data presents logistical, ethical, and privacy challenges [147]. Existing multimodal medical data sets available for public use [84,85,148] are often relatively small in scale and demand the consolidation of numerous resources. For instance, the MIMIC-IV [148] includes a limited range of modalities, including clinical notes, medical images (chest x-ray Digital Imaging and Communications in Medicine [DICM] images), and time series (diagnostic electrocardiogram and patient records), making it a valuable but constrained resource for training medical M-LLMs. Similarly, PMC-VQA [85] and LLaVA-Med [84] include text and image modalities for medical visual question answering.

It is to be noted that the storage of vast amounts of multimodal data (ie, medical images and scans, videos, and high-resolution audio files) requires substantial storage capacity. Efficient and secure storage solutions are essential to handle these data, ensuring quick access and retrieval while maintaining data integrity and security.

##### Potential Solution

To address the limited data challenge in training medical M-LLMs, a combination of synthetic data generation and federated learning could be used. Synthetic data generation using generative models can create realistic, diverse data sets that mimic real-world multimodal medical scenarios, thus expanding the training data set without compromising privacy or ethical standards [149-151]. In addition, federated learning presents a viable solution for leveraging multimodal data from multiple health care institutions without the need to share the actual data, thus maintaining patient privacy [152-156]. This decentralized approach enables multimodal M-LLMs to learn from a vast, distributed data set encompassing a wide range of medical modalities without necessitating centralization of the data.

The few-shot learning approach enables models to generalize from a limited number of examples. By leveraging the pretrained knowledge and adapting quickly to new tasks with minimal data, few-shot learning can be particularly useful in medical scenarios in which labeled data are limited. Another approach to reducing computational requirements and addressing the problem of unavailable labeled data is in-context learning. This approach enables models to perform tasks by providing examples in the input context without fine-tuning the model weights. This approach can be effective for tasks such as medical image interpretation or clinical note analysis.

To address data storage demands when building M-LLMs, cloud-based storage solutions offer a flexible and scalable way to store big data and allow organizations to scale their storage capacity as needed without the upfront investment in physical infrastructure. Other benefits include improved accessibility and cost efficiency, whereas providers can implement robust security measures (eg, data encryption and access control). Moreover, the combination of cloud-based storage and distributed storage systems provides a robust and adaptable solution for managing the extensive and complex data sets needed for M-LLMs.

#### Scalability and Computational Demands

##### Problem

The development and deployment of M-LLMs in the medical field pose significant scalability and computational challenges. During training, such complex M-LLMs require substantial computational power, often involving parallel processing and sophisticated algorithms to manage and analyze the data effectively. Moreover, M-LLMs face memory limitations due to processing vast amounts of data, and their large size necessitates considerable storage capacity. This can also lead to network latency, slowing down model performance and affecting user experience. The scalability issue is further compounded by the need for continuous model updates to incorporate new medical data and knowledge. These factors translate to high operational costs, making the development of medical M-LLMs feasible mainly for large technology corporations with significant resources. Inference, on the other hand, requires minimizing latency and reducing computational load to ensure real-time or near–real-time responses in clinical settings. Both phases pose unique challenges that need to be addressed to facilitate the practical deployment of M-LLMs in health care.

##### Potential Solution

To optimize efficiency during both training and inference, several methods can be used. Parameter-efficient fine-tuning methods such as adapter layers help reduce the computational load by fine-tuning only a subset of the model’s parameters [157,158]. In addition, quantization approaches can address the scalability and computational demands by shifting toward quantized versions of existing models using curated, domain-specific data rather than pretraining from scratch [159]. This method capitalizes on the foundational strengths of established models, significantly reducing the computational resources needed for initial training [160]. Knowledge distillation is another approach that involves training a smaller “student” model to replicate the behavior of a larger “teacher” model, requiring less computational power while retaining performance [161]. Fine-tuning using targeted medical data sets enhances accuracy and relevance in medical applications while also cutting down development time and costs. Furthermore, developing more efficient transformer architectures tailored for multimodal data, such as Kosmos-1 [162], Muse [163], and PixArt-α [164], presents a viable solution. Optimizing algorithms for parallel processing is another approach that promotes more efficient use of computational resources. During inference, quantization and pruning continue to be beneficial by reducing the computational burden and speeding up model execution. Knowledge distillation allows for the use of smaller, faster models that maintain high performance, ideal for real-time applications. Additional optimization techniques, such as model compression [165] and hardware acceleration using graphics processing units (GPUs) or tensor processing units (TPUs) [166], further enhance efficiency.

#### Model Interpretability

##### Problem

In contrast to unimodal LLMs, the scale of M-LLMs in terms of parameters and training data introduces a unique set of interpretability challenges alongside potential opportunities in the field of research on model explainability. First, as these models expand in size, the task of understanding and interpreting their decision-making processes becomes increasingly challenging [167]. This difficulty is amplified by the added internal complexity of M-LLMs and the extensive variety of their training data sets. Moreover, this complexity necessitates substantial computational resources to facilitate the generation of explanations. Such increased complexity poses significant hurdles for in-depth analysis, thereby hindering the debugging and diagnostic processes essential for understanding and improving M-LLMs.

##### Potential Solution

Addressing these interpretability challenges in the context of health care is critical as clinicians—accountable to patients and regulators—should have a reasonable ability to explain how a complex model assists and makes medical recommendations. Choosing between model performance and interpretability can be problematic and is often down to trust (in model development methods, data, metrics, and outcome data, among other things). This challenge necessitates the development of advanced methods for explaining transformer-based language models [167,168], particularly methods for local explanations, such as feature attribution explanation, attention-based explanation, example-based explanation, and natural language explanation [169-172], and global explanations, such as probing-based explanation, neuron activation explanation, concept-based explanation, and mechanistic interpretability [168,173,174]. In addition, being able to use these explanations is crucial for debugging and improving M-LLMs. An effective approach is the development of integrated explanation frameworks specifically designed for medical M-LLMs that can integrate both local and global explanations. Such frameworks are essential for handling the multimodal nature of medical data, including the combination of textual and imaging information. In addition, incorporating a human-in-the-loop approach, where clinician feedback on the model’s explanations is used for continuous improvement, can significantly enhance the practical utility and trustworthiness of these M-LLM systems in medical settings [167].

### Ethical Challenges

#### Bias and Fairness

##### Problem

The potential for bias represents one of the primary ethical challenges in using M-LLMs in health care. Specifically, in the health care domain, data often exhibit bias due to the uneven distribution of demographic attributes, preconceptions held by health care professionals involved in data collection and interpretation, and the varied academic and experiential backgrounds that influence their perspectives [175-177]. If M-LLMs are trained on patient data that contain biases related to gender, ethnicity, socioeconomic status, or geographic location, they may inadvertently cause biases in their predictions or recommendations [175,178,179]. For example, a recently developed M-LLM, LLaVA [46], when asked to analyze an image featuring 2 Black men and 2 gorillas, erroneously identified one of the men as a gorilla. This error suggests the existence of racial bias within the algorithmic framework of the model [180]. In health care, biased M-LLMs can lead to differential treatment, misdiagnoses, and unequal access to medical resources. For example, an M-LLM analyzing medical images might miss subtle symptoms in darker-skinned individuals due to biases in the training data. One study showed that CNNs, when trained on publicly available chest x-ray data sets, may show a tendency to underdiagnose specific populations, such as individuals from marginalized communities (eg, Black and Hispanic patients), women, and Medicaid recipients [181].

##### Potential Solutions

Mitigating bias and improving fairness within medical M-LLMs necessitates a multifaceted approach centered on 3 pillars: data integrity, model refinement, and comprehensive evaluation [181,182]. Essential to this strategy is the curation of diverse and representative data. This involves compiling multimodal medical data sets that encompass a wide array of demographics, languages, and cultures to ensure balanced representation and guide targeted model fine-tuning efforts [183]. Fine-tuning these models through transfer learning and bias reduction techniques, such as counterfactual data augmentation [184], can effectively minimize patterns of gender, racial, or cultural bias. Furthermore, deploying multiple methods and metrics for evaluation is crucial. These may include human, automatic, or hybrid evaluations alongside metrics such as accuracy, sentiment, and fairness, which provide feedback on bias in M-LLM outputs. Through such rigorous evaluation, biases can be detected and continuously addressed, improving the reliability of M-LLMs. Moreover, incorporating logic-aware mechanisms into medical M-LLMs involves integrating clinical reasoning and decision-making processes into the M-LLMs. This approach promotes the generation of more accurate and less biased outputs by applying medical reasoning to the relationships between data tokens. For instance, logic-aware M-LLMs can differentiate between correlational and causal relationships in patient data, recognize the significance of laboratory values within clinical contexts, and apply diagnostic criteria accurately across diverse patient populations. Ultimately, the goal is to reduce bias without compromising the performance of M-LLMs. It is a careful balance of debiasing and enhancing the models, requiring ongoing monitoring and adjustment to align with ethical standards, particularly in the sensitive domain of health care [185].

#### Informed Consent

##### Problem

Obtaining informed consent in the context of M-LLMs presents unique challenges. In particular, it remains uncertain whether patient consent is necessary for training M-LLMs using their data if consent was previously obtained for research purposes in general or for AI development specifically [178,186]. Furthermore, given the complexity of M-LLMs, it might be difficult for patients to grasp what they are consenting to, especially in terms of how their data will be used, how these models operate, and the potential risks involved. This raises questions about the validity of consent and the level of detail required to adequately inform patients [177,178]. In addition, it can be argued that traditional institutional review boards (IRBs) and ethical oversight committees may be ill-equipped to deal with AI and M-LLM applications due to the lack of understanding of such novel technologies in the medical arena [187].

##### Potential Solutions

Health care providers and developers have a responsibility to empower patients to make informed decisions about the use of their data in developing M-LLMs. This requires providing them with clear, transparent, simplified explanations of how M-LLMs work, how their data will be used, the nature of the data they handle, the steps taken to protect privacy, and the potential risks of using their data (eg, algorithmic bias and privacy issues). These explanations may take various forms, including written text, visual aids, educational videos, or other materials tailored to different levels of understanding. Professional training should be provided to health care professionals on the capabilities, limitations, and ethical considerations of using M-LLMs in practice to effectively communicate these aspects to patients. To this point, it may be necessary for health care and academic medical institutions to adapt their IRBs for a more effective governance and use of AI, first through incorporating a sufficiently diverse set of expert members (eg, experts in machine learning, experts in data science, and experts in previous studies of marginalized or discriminated communities) and, second, through more targeted, ongoing training of board members. In doing so, IRBs are more likely to constructively navigate issues pertaining to informed consent, data privacy and security, and safety.

#### Data Privacy and Security

##### Problem

As mentioned previously, M-LLMs require a massive amount of patient data (eg, medical history, clinical notes, medical images, laboratory test results, and prescriptions) that are inherently sensitive. This, in turn, raises substantial privacy and security concerns—how will patient data be collected, stored, and used? Who will have access to them and for what purposes [175-177]? Researchers have demonstrated that bombarding an LLM with specific questions (ie, adversarial attacks) could force it to expose its training data, which contain verbatim personal identifiable information and chat conversations [188]. They have also concluded that larger models seem to be more susceptible to attacks than smaller models [188]. Other studies have shown that, even when sensitive patient data are anonymized, certain algorithms can still identify individual patients [189-191]. Unauthorized access or breaches can have severe consequences, including reputational damage, misuse of personal health information, and compromise of patient confidentiality.

##### Potential Solutions

It is crucial to implement stringent data protection measures to mitigate data privacy and security concerns when using patient data for developing M-LLMs. One of these measures is the implementation of federated learning techniques [153,155,156] to enable M-LLMs to be trained on decentralized data sources without the need to transfer sensitive or private information to a central location, thereby preserving data privacy and security. Furthermore, robust encryption protocols and anonymization techniques should be applied to the data before transferring or processing them. Secure storage infrastructure should be in place to safeguard patient information. It is important to conduct auditing of M-LLMs using data extraction attacks to understand how well M-LLMs resist unauthorized attempts to extract data and identify areas for improvement in terms of security and privacy. Health care providers and developers must establish strong data governance frameworks and policies and comply with relevant privacy regulations (eg, Health Insurance Portability and Accountability Act [HIPAA]). They also need to adopt a proactive approach to cybersecurity and regularly update security measures to counter-emerging threats.

#### Safety and Alignment

##### Problem

Ensuring the safety and alignment of M-LLMs in health care is paramount. These models must not only be effective in processing and analyzing medical data but also align with human ethical standards, particularly those of health care professionals. Similar to text-based models, where fine-tuning, reinforcement learning from human feedback, and dynamic policy optimization (DPO) are used to minimize harm and align outputs with human preferences, M-LLMs could adopt analogous methodologies to ensure that their recommendations are in harmony with the preferences and ethical considerations of medical practitioners. The challenge lies in aligning M-LLMs with the complex, nuanced, and sometimes subjective decision-making processes of human physicians. This involves training models on a diverse array of scenarios, encompassing ethical dilemmas, treatment preferences, and patient-centered care principles. By integrating feedback loops in which health care professionals review and adjust model outputs alongside technical and other professionals, M-LLMs can learn to prioritize patient safety, privacy, and the nuances of human empathy and ethical considerations in their recommendations.

##### Potential Solutions

Developing a framework for continuous learning and adaptation is crucial. This could involve iterative cycles of feedback and adjustment in which M-LLMs are fine-tuned based on direct input from health care professionals regarding the appropriateness and ethical alignment of their outputs. Incorporating mechanisms for DPO in which models adjust their decision-making strategies in real time based on new information or feedback could further enhance alignment with human values. Moreover, simulating diverse clinical and ethical scenarios during training phases can prepare M-LLMs to handle real-world complexities.

## Future Outlook

### Overview

**Figure 6:**
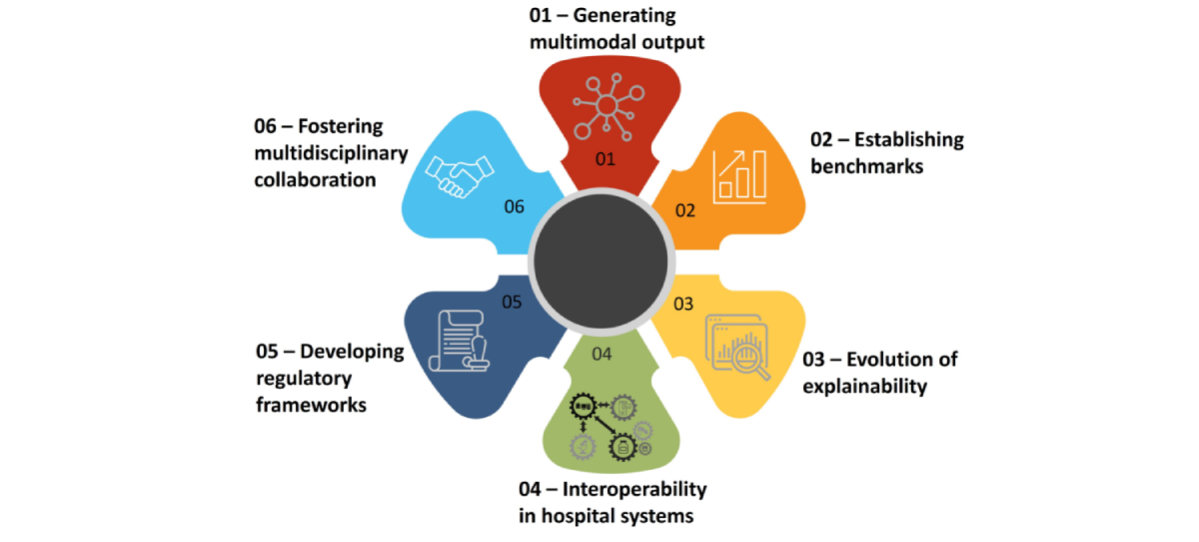
Future directions of multimodal large language models in health care.

In the evolving landscape of medical M-LLMs, anticipating future directions is crucial for advancing their application in health care. In this section, we outline prospective advancements and necessary adaptations that could enhance the functionality, efficacy, and ethical integration of M-LLMs in health care. Specifically, we explore the evolution in generating multimodal outputs, the critical need for establishing performance benchmarks, the shift in explainability paradigms toward comprehensive explainability, the role of M-LLMs in enhancing interoperability within hospital systems, the formulation of robust regulatory frameworks, and the essential role of multidisciplinary collaboration (Figure 6). We envision that these areas collectively represent key future perspectives where M-LLMs are expected to transform both medical applications and patient care.

### Generating Multimodal Outputs

While medical M-LLMs are rapidly evolving in processing multimodal inputs, the development of multimodal outputs is still trailing behind. The importance of multimodal outputs in medical contexts is significant. For example, when asking ChatGPT to explain complex medical concepts, such as interpreting radiological images or outlining surgical procedures, effective explanations should ideally blend textual descriptions with graphical representations, mathematical equations, audio narratives, or animations for enhanced comprehension. This highlights the need for medical M-LLMs capable of producing such varied outputs. A critical step toward this goal is the creation of a shared intermediate output by the model, which raises the following question: what form should this intermediate output take? A practical method is using text as the intermediate output, serving as a basis for generating additional modalities. For example, the causal masked multimodal (CMM) model [192] produces HTML markup that can be transformed into rich web pages with text, formatting, links, and images. Alternatively, using multimodal tokens where each token is tagged to represent different modalities such as text or image offers another route. Image tokens could feed into an image generation model such as a diffusion model to generate visual content, whereas text tokens are processed by a language model. This dual-token approach paves the way for more sophisticated and contextually appropriate multimodal outputs. Further exploration and development in this field could lead to models that seamlessly integrate a variety of output formats, revolutionizing the way in which medical information is conveyed and understood.

### Establishing Benchmarks

Benchmarks are crucial in assessing the performance, accuracy, and effectiveness of generative AI, especially in the context of medical M-LLMs. The expansive scope and complex nature of health care and medicine necessitate continuous advancements in robust evaluation methods and frameworks. This is essential to ensure that medical M-LLMs are effectively aligned with the unique requirements of these domains. These benchmarks enable model comparisons, highlighting efficiencies and creative capabilities in specific tasks and data modalities both individually and collectively. They also play a critical role in detecting biases and limitations. Furthermore, they play a crucial role in establishing industry standards for medical M-LLMs, ensuring their ethical and safe use in sensitive medical contexts. Recent initiatives in M-LLM benchmarks, such as AesBench [193], Mementos [194], MME [195], MM-BigBench [196], MLLM-Bench [197], and VLM-Eval [198], offer a foundational framework that could be adapted to medical M-LLMs. However, there is an urgent need for more comprehensive evaluation methods and frameworks as well as rigorous rubrics for human evaluation of M-LLM performance in real-world clinical workflows and scenarios.

### Evolution of Explainability: From Snapshot to Temporal Explainability

Snapshot explainability refers to the ability of M-LLMs to provide explanations for decisions or predictions at a single, specific point in time. In contrast, temporal analysis offers a more comprehensive understanding by tracking and interpreting changes over time. Most current interpretability research on M-LLMs neglects training dynamics, focusing mainly on post hoc explanations of fully trained models [167]. This lack of developmental investigation into the training process can lead to biased explanations. Moreover, examining interpretability based on a single data modality fails to reflect interactions between modalities. Therefore, transitioning from static snapshot explainability to dynamic temporal analysis is essential for medical M-LLMs. This approach is particularly beneficial for using multimodal data in monitoring patient progress, understanding disease trajectories, and predicting outcomes. By leveraging temporal explainability, M-LLMs can better contextualize data, uncovering patterns and trends that might be overlooked in static analysis. This shift not only enhances the accuracy of diagnoses and treatment plans but also improves the personalization of patient care by taking advantage of rich multimodal data.

### Interoperability in Hospital Systems

An M-LLM could act as a central hub in hospitals, integrating various unimodal AI systems such as radiology, insurance, and EHRs. Currently, each department uses different AI tools from various companies, and most of these systems do not intercommunicate, resulting in access being limited to only department-specific systems. For instance, radiologists use radiological AI, whereas cardiologists might not have access to this, and likewise for other specialties. The introduction of M-LLMs can change this landscape significantly. M-LLMs understand the language and format of all these disparate software applications, allowing for seamless interaction. This means that health care practitioners regardless of specialty could easily work with any AI tool in the hospital, breaking down the silos that currently exist. This potential is vital as it enables comprehensive, integrated care, which individual organizations cannot achieve alone due to proprietary restrictions on data.

### Developing Regulatory Frameworks

The development of a regulatory framework for medical M-LLMs is essential to ensure their safe, effective, and ethical use. Regulatory bodies need to establish standards and guidelines that define acceptable accuracy for various M-LLM applications, ensuring that these tools are reliable and trustworthy in clinical settings. A critical aspect of this framework also includes algorithmic transparency; therefore, regulatory guidelines must clearly stipulate requirements for explainability. Furthermore, the protection of patient data privacy is essential given that M-LLMs process sensitive health information. Therefore, regulatory frameworks must enforce strict data protection standards and formulate strategies for ethically collecting and processing multimodal data sets. Moreover, regardless of whether regulations are sufficiently developed or comprehensive in any given jurisdiction, medical and research institutions have an obligation to upgrade the knowledge and diversity of their ethics approval boards.

### Fostering Multidisciplinary Collaboration and Stakeholder Engagement

AI, and specifically M-LLMs, is so new and complex in the health care domain that the expertise and insights needed extend far beyond the capabilities of any one health care or academic medical organization. Thus, it is imperative for those implementing M-LLM solutions to draw upon the know-how of 4 major external stakeholders. First, because many AI projects are expected to pose ethical concerns, the relevant applicable regulatory bodies and local health authorities should be engaged on a regular basis to ensure compliance with regulations. Indeed, guidelines and laws are rapidly changing; at the time of writing, the European Union has endorsed a world-first AI Act [199]. Second, much of the M-LLM innovation is expected to stem from academic and research contexts, where scientists continually push the boundaries of evidence-based, validated AI projects commonly published and made available for public benefit. Collaborating and partnering with such institutions ensures that the latest approaches and technologies can be incorporated into a health care project. Third, the industry is often a forgotten collaborator due to perceived entry barriers (eg, intellectual property ownership, exclusivity, and so forth). However, large commercial companies have access to far wider resources and technical expertise, particularly in engineering development, than medical institutions and, when negotiated with a win-win perspective, can significantly accelerate AI project deployment in the health care context. The same may apply to vendors who are infrastructure and deployment experts and who may be able to contribute beyond the limited scope of a purchase agreement. Moreover, when applicable, industry partners may offer greater commercialization pathways for projects. Finally, the fourth external stakeholder is the patient advocacy organization. Such groups should be engaged early and continuously and can help ensure that patients’ critical perspectives are communicated and included within the requirements of an M-LLM project. This is especially the case in projects that directly impact the patients’ needs and preferences, for instance, an M-LLM that interacts by providing clinical insights and recommendations to the physician during a patient consultation. Such advocacy groups can also be an effective way for health care institutions to more naturally engage in awareness and trust building with their communities. Naturally, with external stakeholders, appropriate collaboration and data agreements should be sought to protect the health care institutions’ interests as well as those of their patients. In addition, regardless of whether projects require internal or external collaboration, best practices should be used to ensure that roles, responsibilities, and decision-making structures are clarified upfront.

## Conclusions

In this paper, we explored the foundational principles, applications, challenges, and future perspectives of M-LLMs in health care practice. While this work suggests a promising direction for the application of M-LLMs in medicine, it also highlights the need for further evaluation and benchmarking of their capabilities and limitations in real-world medical settings. In addition, despite the momentum toward models capable of processing multimodal inputs, the progression toward sophisticated multimodal outputs remains comparatively slow. Furthermore, it is crucial to acknowledge that the emergence of M-LLMs does not render traditional LLMs obsolete. Instead, M-LLMs serve as an extension, building upon the foundational strengths and capabilities of LLMs to enhance health care delivery. This association underscores that the efficiency of M-LLMs is inherently tied to the robustness of the underlying LLMs. As we advance toward more general AI systems, M-LLMs offer a promising path to a comprehensive form of AI in health care practice. The journey has its challenges, but the potential rewards could significantly redefine our interaction with technology in the medical field.

## Abbreviations

AGI: artificial general intelligence
AI: artificial intelligence
BERT: Bidirectional Encoder Representations From Transformers
CGM: continuous glucose monitoring
CMM: causal masked multimodal
CNN: convolutional neural network
CONCH: Contrastive Learning From Captions for Histopathology
COPD: chronic obstructive pulmonary disease
CT: computed tomography
DICM: Digital Imaging and Communications in Medicine
DPO: dynamic policy optimization
EHR: electronic health record
GPT: generative pretrained transformer
GPU: graphics processing unit
H&E: hematoxylin and eosin
HIPAA: Health Insurance Portability and Accountability Act
IRB: institutional review board
LLM: large language model
LSTM: long short-term memory
M-LLM: multimodal large language model
MLM: mask language modeling
MRI: magnetic resonance imaging
NLP: natural language processing
RNN: recurrent neural network
TPU: tensor processing unit
